# Clinical efficacy of fire-needle warming therapy in the treatment of knee osteoarthritis of cold-dampness type and its effect on serum IL-1β and MMP-3

**DOI:** 10.1007/s10067-025-07497-7

**Published:** 2025-06-10

**Authors:** Tanshu Liu, Qingqing Lian, Yuan Zeng, Binfu Que, Yonglong Tang, Wenbao Wu, Rui Qiu

**Affiliations:** Department of Acupuncture and Moxibustion, Longyan First Hospital Affiliated to Fujian Medical University, No. 105, Jiuyi North Road, Longyan City, 364000 Fujian Province China

**Keywords:** Clinical efficacy, Cold-dampness arthralgia type knee osteoarthritis, Fire-needle warming method, IL-1β, MMP-3

## Abstract

**Objective:**

This study aims to explore the clinical efficacy of fire-needle warming therapy in managing knee osteoarthritis attributed to cold-dampness patterns. Specifically, it evaluates the therapy’s influence on interleukin-1β (IL-1β) serum concentrations and matrix metalloproteinase-3 (MMP-3).

**Methods:**

A retrospective analysis was conducted on 80 patients treated for knee osteoarthritis from September 2023 to February 2024. Patients were divided into two groups: 40 received electroacupuncture, and 40 received fire acupuncture combined with warming techniques. The primary outcome measures included treatment efficacy, Western Ontario and McMaster Universities Osteoarthritis Index (WOMAC) scores, compare the changes in IL-1β and MMP-3 levels in serum and synovial fluid between two groups, and compare the total knee joint X-ray scores between the two groups: pain levels and knee joint function. Adverse reactions were also recorded.

**Results:**

The fire acupuncture group had a significantly higher treatment efficacy rate (92.5%) compared to the electroacupuncture group (75%, *P* < 0.05). Both groups showed significant reductions in WOMAC scores post-treatment, with a greater reduction in the fire acupuncture group (*P* < 0.05). Serum levels of IL-1β and MMP-3 decreased significantly in both groups, with a more pronounced decrease in the fire acupuncture group (*P* < 0.05). After 4 weeks of intervention and 8 weeks of follow-up, the levels of IL-1β and MMP-3 in the synovial fluid of the fire needle group and the electroacupuncture group were significantly reduced (*P* < 0.05), and the fire needle group was significantly lower than the electroacupuncture group (*P* < 0.05). Pain and knee function scores improved significantly in both groups, with the fire acupuncture group showing greater improvements (*P* < 0.05). After 4 weeks of intervention and 8 weeks of follow-up, the total joint X-ray scores of the fire needle group and the electroacupuncture group were significantly reduced (*P* < 0.05), and the fire needle group was significantly lower than the electroacupuncture group (*P* < 0.05). The adverse reaction rate was lower in the fire acupuncture group (5%) compared to the electroacupuncture group (20%, *P* < 0.05).

**Conclusions:**

Fire-needle warming therapy demonstrates significant clinical efficacy in treating knee osteoarthritis of the cold-dampness type. It effectively reduces inflammation and pain and improves knee function, suggesting its potential for broader clinical application. Further research is recommended to confirm these findings.

**Table:** 

**Key Points** • *Fire needle acupuncture for knee osteoarthritis: Fire needle acupuncture is highly effective in treating cold-dampness arthralgia-type knee osteoarthritis by promoting blood circulation, enhancing metabolism, and reducing inflammation.* • *Clinical and biochemical outcomes: Patients receiving fire needle acupuncture showed significantly better clinical outcomes, including lower pain scores, improved knee function, and decreased serum levels of IL-1β and MMP-3 compared to those receiving electroacupuncture.* • *Safety and efficacy: Fire needle acupuncture provides superior therapeutic effects and results in fewer adverse reactions compared to electroacupuncture, highlighting its safety and efficacy in clinical practice.*

## Introduction

Osteoarthritis of the knee is a widespread ailment known for the progressive deterioration of joint cartilage, predominantly exhibiting as pain and compromised functionality within the joint [1]. As the world’s population ages and obesity increases, rate worldwide occurrence of knee osteoarthritis shows an obvious upward trend, Not only does this health issue exacerbate the personal and familial health and economic burdens, but it also detrimentally affects the national healthcare system, thereby driving up the societal and economic costs [2]. While nonsteroidal anti-inflammatory drugs (NSAIDs) are frequently used to treat knee osteoarthritis, they are associated with various side effects. The study compared the efficacy of fire acupuncture, warm acupuncture and moxibustion, electroacupuncture, and Western medicine (including NSAIDs) in the treatment of knee osteoarthritis through mesh meta-analysis. The results show that fire needles have advantages in improving WOMAC stiffness. In terms of reducing the total score of WOMAC, fire acupuncture, warm acupuncture and moxibustion, and electroacupuncture are superior to Western medicine [3]. Thus, it is crucial to identify treatments that are both safer and more efficacious. In traditional Chinese medicine, 0ngs to the category of “Bi syndrome,” and more emphasis is placed on the importance of symptomatic treatment [4]. Main pathological characteristics of cold-dampness arthralgia type knee osteoarthritis are as follows [5, 6]: the local cold-damp environment in the joint is obvious, resulting in symptoms such as poor circulation of Qi and blood, joint pain, stiffness, etc.; severe inflammatory reaction in the joint synovium; serious wear of articular cartilage, narrowing of the joint space, and even joint bone ankylosis. The key to the treatment of this type of disease is warmth. It can dissipate yang and dampness, unblock collaterals to relieve pain, anti-inflammatory, and reduce swelling. Moisture, as an exogenous evil, significantly influences the pathogenesis of bone and joint disorders [7]. Fire needle acupuncture, known for its warming, blood-activating, and pain-relieving properties, significantly aids in treating arthritis and can slow its progression [8]. Its principle of action is mainly reflected in the following aspects [9, 10]: the high temperature of fire needles can stimulate acupuncture points and stimulate meridian qi, thus dredging the meridians and mediating qi and blood; the warming effect of fire acupuncture can accelerate local blood circulation, improve local tissue hypoxia and malnutrition, and accelerate the absorption and metabolism of inflammatory substances; fire acupuncture therapy can also achieve anti-inflammatory and analgesic effects by regulating the nervous system, immune system, etc., immune regulation, and other effects. In traditional Chinese medicine, the warm-tonifying method is employed for treatment, which mainly treats diseases by warming yang, reducing dampness, unblocking collaterals, and relieving pain. For the treatment of knee osteoarthritis with characteristics of cold-dampness arthralgia, the mechanism of action of warming method mainly includes the following points [11, 12]: first, it warms yang and removes dampness, improving the local cold and damp environment of the joints which allows Qi and blood to flow smoothly; the second is to unblock meridians and relieve pain, and relieve joint pain and stiffness; the third is to promote local blood circulation and reduce joint inflammatory reactions; the fourth is to enhance the body’s immune function and improve disease resistance. The objective is to investigate the clinical benefits of fire acupuncture and warming therapy for managing knee osteoarthritis of the cold-dampness subtype, while also examining the treatment’s effects on interleukin-1β (IL-1β) and matrix metalloproteinase-3 (MMP-3) in the serum. The report is presented below.

## Materials and methods

### Statement of human and animal rights

Approval for the study was obtained from Fujian Medical University Affiliated Longyan First Hospital: Longyan First Hospital of ethics committee.

All of the patients had consented to research authorization for record review, and the study was approved by the institutional review board.

### Clinical data

Eighty patients with knee osteoarthritis who were diagnosed and treated at our hospital were retrospectively selected for research and analysis. The selected time period was from September 2023 to February 2024. Inclusion criteria were as follows: ① patients all meet knee osteoarthritis diagnoses and treatments are in accordance with the traditional Chinese medicine guidelines [13] and Western medicine [14]; ② patients all belong to the cold-dampness type of knee osteoarthritis; ③ no infectious diseases; ④ have not taken glucocorticoids, non-steroidal anti-inflammatory drugs, and other drugs that may affect the results 2 months before participating in the study. Exclusion criteria were as follows: ① patients with impaired liver, renal, or cardiac function; ② patients with severe allergies; ③ patients with rheumatoid arthritis; ④ patients who cannot cooperate well with the examinations in the study. All eligible patients were categorized into two distinct groups based on the therapeutic approaches they received: 40 patients in the electroacupuncture group receiving conventional electroacupuncture treatment and 40 patients in the fire needle group receiving fire needle warming therapy. Among them, the patients in the electroacupuncture group were 40–75 years old, with an average age of 59.88 ± 8.13 years; there were 25 males and 15 females; the illness duration was 1.5–3.1 years, with an average disease duration of 2.85 ± 0.74 years. The age of the observation group was 42 ~ 75 years old, with an average age of 61.27 ± 11.15 years; there were 17 females and 23 males; the illness duration was 1.5 ~ 3.1 years, with an average disease duration of 2.79 ± 0.89 years. The two groups did not differ significantly in their general information (*P* > 0.05). This study was approved by the Hospital Ethics Committee (Approval Number: LYREC2023-k072-01).

### Method

Fire acupuncture group: (1) Basis for acupoint selection and acupoint positioning: refer to the acupoint selection requirements for cold-dampness type knee paralysis in the planning textbooks “Acupuncture and Moxibustion Therapeutics” and “Meridian and Acupoint Science” in colleges and universities of traditional Chinese medicine across the country and combined with the many years of use of fire acupuncture in our department. Wen Tong method is used to treat KOA based on experience in acupoint selection: inner knee eye (EX-LE4), outer knee eye (EX-LE5), Yinlingquan (SP9), Yanglingquan (GB34), Xuehai (SP10), Liangqiu (ST34), Yaoyangguan (DU3), Ashi acupoint. (2) Operation method: Before the fire acupuncture treatment, the patient should do sufficient ideological work to relieve the fear in his mind, so that he can better cooperate with the treatment when the doctor performs the surgery. During the operation, the patient should be in a supine or sitting position, and the affected knee should be fully exposed. Before the fire needle operation, 75% alcohol should be used for routine disinfection. The doctor chooses Liu Enming’s medium fire needle (specification: 0.5 mm × 25 mm) and uses the right thumb 1. Hold the needle handle of the fire needle with your index finger and hold the alcohol lamp with your left hand. Burn the needle body in the outer flame of the alcohol lamp until it turns white. Take the needle from each acupoint and quickly insert it. After inserting the needle, do not lift or twist the needle and quickly withdraw the needle. The needle is controlled by wrist force, the time is about 0. 5 s, and the operation process requires “steady, accurate, and fast.” After fire acupuncture treatment, the patient is instructed not to wash the needle site that day and avoid getting wet. Treatment is given once every Monday and Thursday for 4 consecutive weeks. After fire acupuncture treatment, the patient is instructed not to scratch and avoid local contact with water to prevent infection.

Electroacupuncture group: The acupoint selection and body positions are the same as those of the fire acupuncture group. Before the operation, explanations should also be done to eliminate the patient’s psychological fear. First, 75% alcohol is used for routine disinfection. The doctor uses Huatuo brand for routine local skin disinfection—disposable sterile acupuncture needles (Suzhou Medical Supplies Factory Co., Ltd. specifications: 0.30 m). The needles are inserted vertically into Ling, Xuehai, Yaoyangguan, and Ashi points, and the depth of needle insertion is 20–25 mm. After inserting the needles, lift, insert, twist, flatten, flatten, and reduce to get Qi, and then use Huatuo brand SDZ-II electrotherapy acupuncture treatment instrument, pair the electroacupuncture. The above acupuncture points are Liangqiu with Xuehai, inner knee eye with Dubi, Yinlingquan with Yanglingquan, Ashi point, etc. Select continuous wave and adjust the acupuncture current intensity. Adjust to the intensity that the patient can tolerate, and leave the needle in place for 30 min. Treatment is given once every Monday and Thursday for 4 consecutive weeks.

### Observation indicators

1. Clinical effect: Evaluate the clinical effect of the patient, including clinical control (limb joint pain, numbness, and other symptoms disappear, TCM syndrome points are reduced by more than 95%, and daily activities have no significant impact), significant effect (symptoms are significantly improved, TCM syndrome points are reduced by 40 to 95%, joint activities are significantly improved), and ineffective (symptoms are not significantly improved, TCM syndrome points are reduced by less than 40%, joint limitations are severe), and the treatment effectiveness is calculated.
2. Serum IL-1β and MMP-3: Take 4 mL of fasting venous blood from patients before treatment, four courses of treatment, and 8 weeks of follow-up at the end of treatment. Collect the supernatant and employ ELISA to assess the patient’s serum IL-1 levels pre-treatment, at 4 weeks post-treatment, and at 8 weeks post-treatment. Utilize Excel to analyze the levels of MMP-3 before and after the treatment, plot a standard linear regression curve with the standard concentration as the *x*-axis and the corresponding OD value as the *y*-axis. Calculate the concentration values of each specimen according to the curve equation.
3. Synovial fluid IL-1β and MMP-3: 5 mL of knee synovial fluid was collected from two groups of patients before treatment, after four treatment courses, and at 8-week follow-up after treatment. Enzyme-linked immunosorbent assay was used to detect the levels of IL-1β and MMP-3 in the synovial fluid.
4. The internationally recognized WOMAC: WOMAC was utilized to assess knee joint function before and after treatment, as well as during follow-up. The WOMAC score consists of a total of 24 items. There are five items, two items in the joint stiffness part, and 17 items in the joint function part. Each item receives a score between 1 and 4 points, culminating in a maximum score of 96; an elevated score corresponds to a poorer knee joint functionality.
5. Assess the patient’s pain using the VAS, rated from 0 to 10, where a higher score indicates greater pain intensity. For knee joint functionality, employ the HSS score, comprising six items with a maximum of 100 points, where a higher score denotes better function. Additionally, use the Lequesne Index, which includes six items and a maximum of 21 points, with higher scores reflecting worse knee function.
6. Knee joint X-ray score: Three symptoms of joint pain, morning stiffness, and swelling were included in the study. According to the severity, from no, mild, moderate to severe, the knee joint X-ray score was expressed as 0, 1, 2, and 3 points, respectively. The higher the score, the worse the knee joint function of the patient.
7. Adverse reactions: Observe the occurrence of adverse reactions during treatment, including pain, scars, and bleeding.

### Statistical methods

Data were analyzed using SPSS 21.0 statistical software. In this study, the measurement data between the two groups were compared using the independent sample *t* test, with $(\overline{x}\pm{s})$ indicates that the indicators include IL-1β, MMP-3, VAS score, HSS score, and Lequesne score. In the study, the count data between groups were contrasted using the *χ*^2^ test, expressed as cases (%), and the indicators include clinical effects, adverse effects, and reaction incidence. Statistical results considered *P* < 0.05 as an outstanding statistical discrepancy.

## Results

### Analysis of clinical effects of two groups

The efficacy rate of treatment in the electroacupuncture group was 75.00% (clinical control 42.50% + effective 32.50%), and the efficacy rate in the fire acupuncture group was 92.50% (clinical control 55.00% + effective 37.50%). The efficacy rate of treatment in the fire acupuncture group was outstanding higher than that of the electroacupuncture group (*P* < 0.05) (see Table 1).

**Table 1:** Analysis and comparison of clinical effects between two groups (cases (%))

Group	Number of examples	Clinical control	Efficient	Invalid	Efficient
Electroacupuncture group	40	17 (42.50)	13 (32.50)	10 (25.00)	30 (75.00)
Fire needle group	40	22 (55.00)	15(37.50)	3 (7.50)	37 (92.50)
*χ*^*2*^					4.501
*P*					0.034

### Analysis of serum IL-1β and MMP-3 in the two groups

**Fig. 1:**
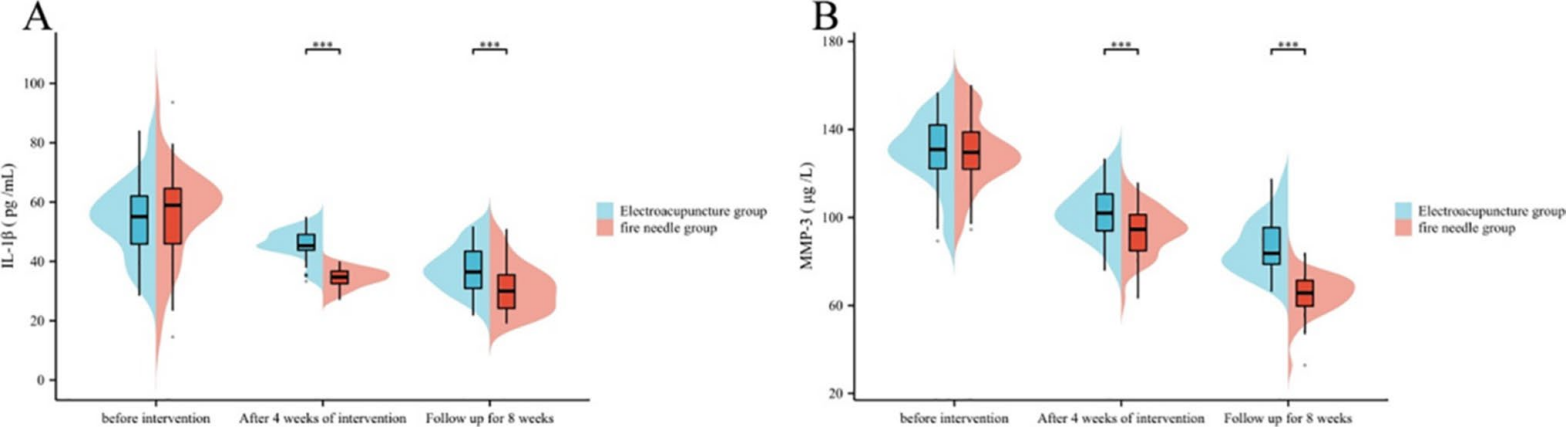
Comparative analysis of serum IL-1β and MMP-3 in the two groups. **A** Comparative analysis of serum IL-1β in the two groups; **B** comparative analysis of serum MMP-3 in the two groups. Compared with the electroacupuncture group after treatment ***P < 0.001

Before the intervention, there was no notable variation in the serum IL-1β and MMP-3 levels between the electroacupuncture group and the fire acupuncture group (*P* > 0.05); after 4 weeks of intervention and 8 weeks of follow-up, the serum IL-1β and MMP-3 levels in the fire acupuncture group and the electroacupuncture group 3 levels were outstanding lower (*P* < 0.05), and the fire acupuncture group was outstanding lower than the electroacupuncture group (*P* < 0.05) (refer to Table 2 and Fig. 1).

**Table 2:** Comparative analysis of serum IL-1β and MMP-3 in the two groups $(\overline{x}\pm{s})$

Group	Number of examples	IL-1β (pg/mL)			MMP-3 (μg/L)		
		Before intervention	After 4 weeks of intervention	Follow-up for 8 weeks	Before intervention	After 4 weeks of intervention	Follow-up for 8 weeks
Electroacupuncture group	40	54.42 ± 12.80	45.45 ± 5.33	37.57 ± 7.58	129.90 ± 15.81	102.68 ± 11.98	86.63 ± 12.30
Fire needle group	40	55.12 ± 15.74	34.25 ± 3.25	30.82 ± 8.28	129.47 ± 15.97	93.25 ± 10.74	64.58 ± 9.27
*t*		0.218	11.347	3.803	0.121	3.707	9.054
*P*		0.828	< 0.001	0.001	0.904	< 0.001	< 0.001

### Analysis of IL-1β and MMP-3 in two groups of synovial fluid

Before intervention, there was no significant difference in the levels of IL-1β and MMP-3 in the synovial fluid between the electroacupuncture group and the fire needle group (*P* > 0.05); after 4 weeks of intervention and 8 weeks of follow-up, the levels of IL-1β and MMP-3 in the synovial fluid of the fire needle group and the electroacupuncture group were significantly reduced (*P* < 0.05), and the fire needle group was significantly lower than the electroacupuncture group (*P* < 0.05) (see Table 3).

**Table 3:** Comparative analysis of IL-1β and MMP-3 in two groups of synovial fluid $(\overline{x}\pm{s})$

Group	Number of examples	IL-1β (pg/mL)			MMP-3 (pg/mL)		
		Before intervention	After 4 weeks of intervention	Follow-up for 8 weeks	Before intervention	After 4 weeks of intervention	Follow-up for 8 weeks
Electroacupuncture group	40	6.14 ± 1.55	4.19 ± 1.85	3.20 ± 0.78	34.58 ± 8.64	29.41 ± 5.97	25.32 ± 4.93
Fire needle group	40	6.22 ± 1.65	2.87 ± 0.74	1.98 ± 0.79	35.62 ± 7.41	25.38 ± 4.62	20.60 ± 3.25
*t*		0.224	4.190	6.950	0.578	3.376	5.056
*P*		0.824	0.001	< 0.001	0.565	0.001	< 0.001

### Comparison of WOMAC scores between two groups

**Fig. 2:**
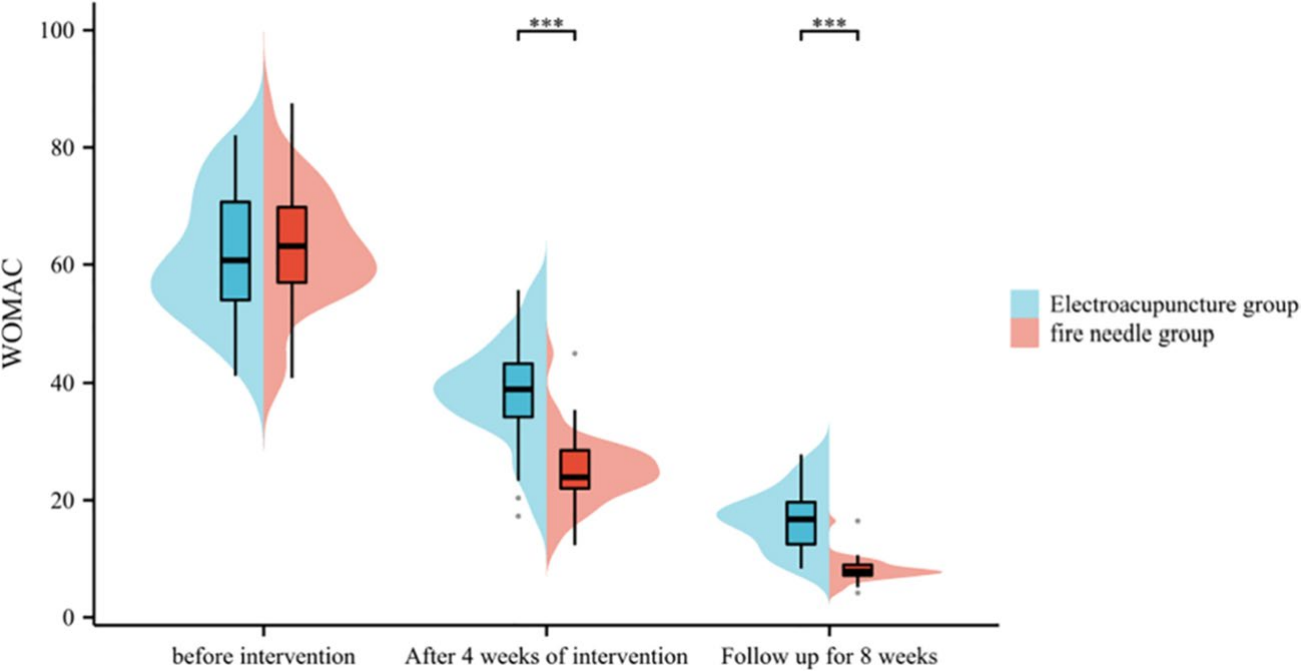
Comparison of WOMAC scores between two groups. Compared with the electroacupuncture group after treatment ***P < 0.001

There was no substantial variation in WOMAC scores between the electroacupuncture group and the fire acupuncture group (*P* > 0.05); after 4 weeks of intervention and 8 weeks of follow-up, the WOMAC scores of both the fire acupuncture group and the electroacupuncture group were significantly reduced (*P* < 0.05), and the fire acupuncture group was outstanding lower than that in the electroacupuncture group (*P* < 0.05) (consult Table 4 and Fig. 2).

**Table 4:** Comparison of WOMAC scores between two groups $(\overline{x}\pm{s})$

Group	Number of examples	WOMAC score (points)		Follow-up for 8 weeks
		Before intervention	After 4 weeks of intervention	
Electroacupuncture group	40	61.25 ± 11.33	38.25 ± 8.74	16.82 ± 10.13
Fire needle group	40	63.42 ± 9.78	24.36 ± 4.89	7.86 ± 6.42
*t*		0.917	8.772	4.725
*P*		0.362	< 0.001	< 0.001

### Pain degree and knee joint function changes of patients in the two groups

**Fig. 3:**
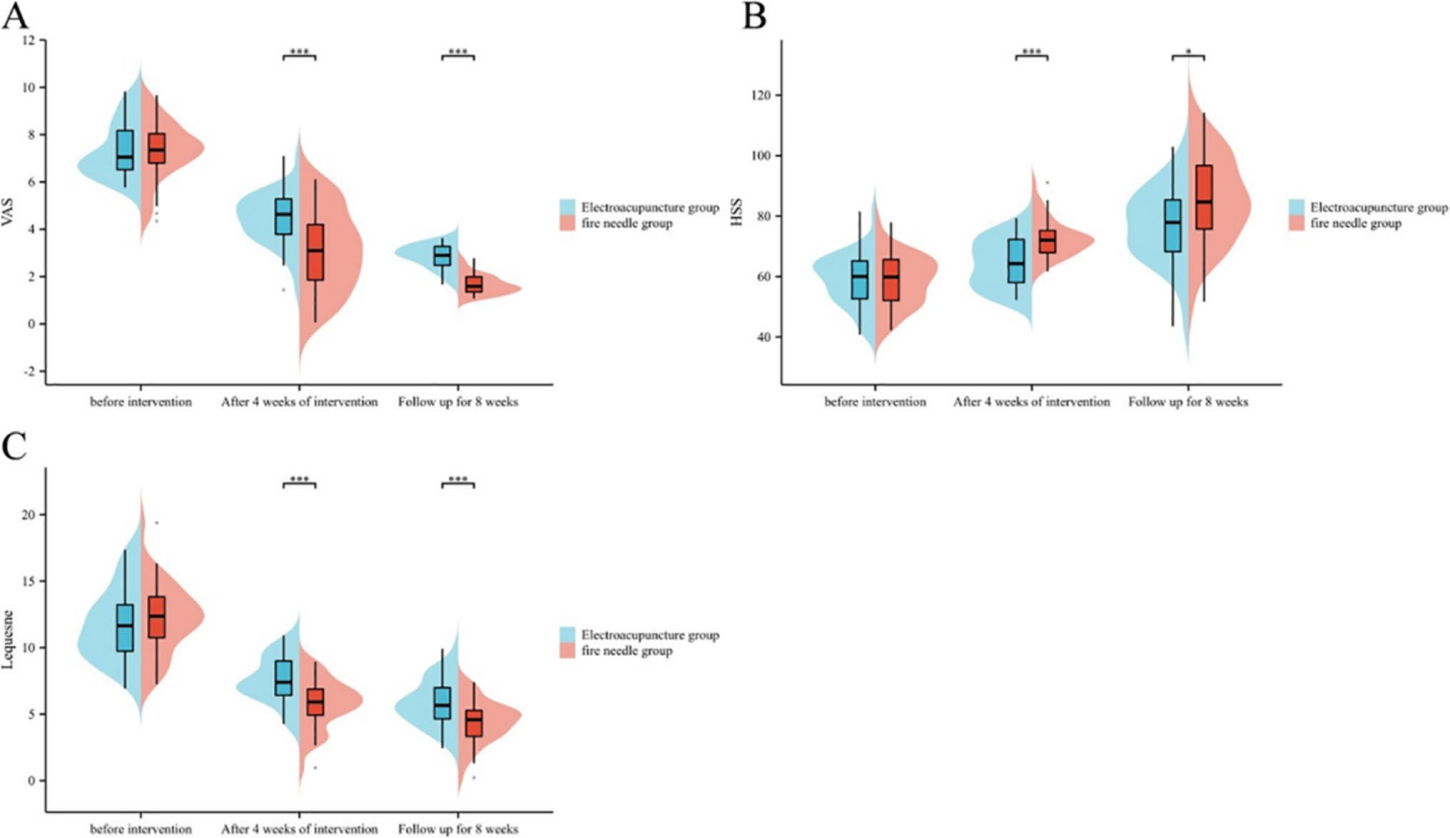
Comparison of pain levels and knee joint function changes between the two groups of patients. **A** Comparison of VAS scores between the two groups of patients; **B** comparison of HSS scores between the two groups of patients; **C** comparison of the Lequesne scores of the two groups of patients. Compared with the electroacupuncture group after treatment ***P < 0.001

Before the intervention, there were no outstanding differences in VAS scores, HSS scores, and Lequesne scores between the electroacupuncture group and the fire acupuncture group (*P* > 0.05); after 4 weeks of intervention and after 8 weeks of follow-up, there were significant differences in VAS scores and Lequesne scores between the fire acupuncture group and the electroacupuncture group decreased (*P* < 0.05), and the fire acupuncture group was outstanding lower than the electroacupuncture group (*P* < 0.05); the HSS scores in both the fire acupuncture group and the electroacupuncture group were outstanding increased (*P* < 0.05), and the fire acupuncture group was outstanding higher than the electroacupuncture group (*P* < 0.05) as shown in Table 5 and Fig. 3.

**Table 5:** Comparison of pain levels and knee joint function changes between the two groups of patients $(\overline{x}\pm{s})$

Score	Time	Electroacupuncture group (*n* = 40)	Fire needle group (*n* = 40)	*t*	*P*
VAS score (points)	Before intervention	7.41 ± 1.18	7.30 ± 1.22	0.410	0.682
	After 4 weeks of intervention	4.45 ± 1.09	3.03 ± 1.72	4.410	< 0.001
	Follow-up for 8 weeks	2.85 ± 0.50	1.64 ± 0.39	12.068	< 0.001
HSS score (points)	Before intervention	58.70 ± 8.64	58.46 ± 8.49	0.125	0.901
	After 4 weeks of intervention	64.25 ± 7.99	72.74 ± 6.40	5.245	< 0.001
	Follow-up for 8 weeks	76.90 ± 12.46	85.47 ± 14.57	2.827	0.006
Lequesne score (points)	Before intervention	11.77 ± 2.74	12.16 ± 2.63	0.649	0.518
	After 4 weeks of intervention	7.59 ± 1.75	5.77 ± 1.87	4.494	< 0.001
	Follow-up for 8 weeks	5.87 ± 1.74	4.41 ± 1.62	3.884	< 0.001

### Improvement of overall joint X-ray scores in two groups

Before intervention, there was no significant difference in the total joint X-ray scores between the electroacupuncture group and the fire needle group (*P* > 0.05); after 4 weeks of intervention and 8 weeks of follow-up, the total joint X-ray scores of the fire needle group and the electroacupuncture group were significantly reduced (*P* < 0.05), and the fire needle group was significantly lower than the electroacupuncture group (*P* < 0.05) (see Table 6).

**Table 6:** Improvement of overall joint X-ray scores in two groups $(\overline{x}\pm{s})$

Group	Number of examples	Joint X-ray overall score (points)		Follow-up for 8 weeks
		Before intervention	After 4 weeks of intervention	
Electroacupuncture group	40	8.05 ± 0.55	6.12 ± 0.98	5.32 ± 0.42
Fire needle group	40	8.20 ± 0.63	5.32 ± 0.67	3.84 ± 0.58
*t*		1.134	4.262	13.071
*P*		0.260	< 0.001	< 0.001

### Comparison of adverse reactions between the two groups of patients

There were five cases of skin pain and three cases of bleeding at the needle eye in the electroacupuncture group, with an adverse reaction rate of 20.00%; there was one case of skin pain and one case of scar at the needle eye in the fire needle group, and the incidence of adverse reactions was 5.00%. The fire needle group was significantly lower than the electric needle group (*P* < 0.05) (see Table 7).

**Table 7:** Comparison of adverse reactions among patients in the two groups (cases (%))

Group	Number of examples	Skin pain at the needle eye	Scar	Hemorrhage	Total
Electroacupuncture group	40	5 (12.50)	0 (0.00)	3 (7.50)	8 (20.00)
Fire needle group	40	1 (2.50)	1 (2.50)	0 (0.00)	2 (5.00)
*χ*^2^					4.114
*P*					0.043

## Discussion

A common ailment, knee osteoarthritis, is a degenerative disease that involves pain in the joints, stiffness, and joint swelling as the main clinical symptoms [15, 16]. Managing knee osteoarthritis typically involves surgical procedures, the administration of drugs, and physical therapy sessions. Among them, pharmaceutical interventions for knee osteoarthritis are largely focused on the use of NSAIDs and intra-articular injections of steroids or sodium hyaluronate. Although it reduces pain and improves mobility to a certain extent, the primary concerns that remain to be tackled are the adverse effects and the high expenses associated with drug treatments [2, 4]. Traditional Chinese medicine emphasizes syndrome differentiation and treatment. It is believed that the primary cause of paralysis is attributed to a deficiency of the liver and kidneys, coupled with an invasion of cold and dampness. Warming the meridians, dispersing cold, and activating blood circulation are the main methods of treatment, and reducing pain and improving function are the main goals of treatment [4]. Acupuncture, a traditional Eastern intervention, is being widely used around the world and is considered an effective and safe analgesic treatment for different types of musculoskeletal pain. Relevant research reports suggest [17] for patients with knee osteoarthritis, acupuncture is key in providing relief from pain and enhancing their ability to function. The findings from this research indicate that the therapeutic effectiveness of the fire acupuncture group was significantly higher than that of the electroacupuncture group. It shows that the fire-needle warming method can have a better effect in treating cold-dampness-induced knee osteoarthritis.

Inflammatory reaction could be implicated in the emergence and development of knee osteoarthritis. IL-1β is considered to be one of the main participants in the inflammatory response. It is mainly produced by chondrocytes, monocytes, osteoblasts, etc., and can provoke the emission of various factors associated with inflammation and tissue breakdown. Various research findings have indicated that the level of IL-1β in synovial fluid in patients with knee osteoarthritis is significantly elevated [18, 19]. Reports indicate that IL-1β not only acts independently but also synergizes with other factors and regulates the synthesis of extracellular matrix components including MMP-3 [20, 21]. MMP-3 is a major enzyme targeting cartilage breakdown. It can degrade type II collagen (CII) and osteonectin (ON) in cartilage, influencing structural alterations in the knee joint [22, 23]. In this study, a comparative analysis of IL-1β and MMP-3 levels before and after the intervention indicated that the fire acupuncture group had significantly lower levels than the electroacupuncture group following treatment. It shows that the fire-needle warming method can efficiently inhibit the inflammatory reaction in patients with knee osteoarthritis. In addition, studies have confirmed that pain is the principal clinical feature of knee osteoarthritis. More than half of patients experience pain symptoms, and more than 25% of patients are accompanied by severe or even unbearable pain, which has a significant impact on knee joint function [24]. The outcomes of this research found that after treatment, the Lequesne score and VAS score of the fire acupuncture group were significantly lower than those of the electroacupuncture group, and elevated HSS scores compared to electroacupuncture. It shows that the fire-needle warming method significantly eases patient pain and enhances knee joint function.

This may be because the fire needle warming method can promote blood circulation to a certain extent, enhance the metabolism of local tissues, improve microcirculation, eliminate patient joint swelling, reduce cartilage damage, thereby alleviate the progression of the disease, reduce pain, and promote functional recovery [25, 26]. Furthermore, the study found that post-treatment, the fire acupuncture group had fewer adverse reactions than the electro-acupuncture group, further confirming the reliability of the treatment method.

## Conclusion

In summary, the fire-needle warming method has a high clinical effect in the treatment of cold-dampness arthralgia type knee osteoarthritis. It can better inhibit the inflammatory reaction, reduce pain, and improve knee joint function. It is useful for guiding clinical treatment of great significance. More patients may benefit from fire-needle warming therapy. However, the data collection period of this study is limited, and the severity of the disease among the incomplete uniformity of the included subjects could have an effect on the reliability of the outcome metrics. In the future, further improvements can be made to address the research deficiencies.

## Data Availability

The datasets used or analyzed during the current study are available from the corresponding author on reasonable request.
